# Isolation, Purification, Fractionation, and Hepatoprotective Activity of Polygonatum Polysaccharides

**DOI:** 10.3390/molecules29051038

**Published:** 2024-02-28

**Authors:** Yutong Wang, Hongmei Niu, Yue Ma, Guangxin Yuan

**Affiliations:** School of Pharmacy, Beihua University, Jilin 132013, China; wangyutong9810@163.com (Y.W.); hongmeiniu@outlook.com (H.N.); my13514344951@163.com (Y.M.)

**Keywords:** Polygonatum polysaccharides, anti-oxidant, anti-inflammatory, hepatoprotective activity

## Abstract

In this study, three homogeneous fractions, PSP-N-b-1, PSP-N-b-2, and PSP-N-c-1, were obtained from an aqueous extract of Polygonatum using DEAE cellulose column chromatography, CL-6B agarose gel chromatography, and Sephadex G100 chromatography. Their monosaccharide compositions and molecular weights were analyzed. The results revealed that PSP-N-b-1, PSP-N-b-2, and PSP-N-c-1 are primarily composed of six monosaccharides: Man (mannose), GlcA (glucuronic acid), Rha (rhamnose), GalA (galacturonic acid), Glc (glucose), and Ara (arabinose), with molecular weights of 6.3 KDa, 5.78 KDa, and 3.45 KDa, respectively. Furthermore, we observed that Polygonatum polysaccharides exhibited protective effects against CCL_4_-induced liver damage in HepG2 cells in vitro, operating through both anti-oxidant and anti-inflammatory mechanisms. Our research findings suggest that Polygonatum polysaccharides may emerge as a promising option in the development of hepatoprotective drugs or functional foods with anti-inflammatory and antioxidant properties.

## 1. Introduction

Liver injury is a complex process resulting from external factors invading the liver, causing damage to its structure and functions. The liver plays a central role in the physiological processes of the human body, participating in the regulation of carbohydrate, lipid, and protein metabolism to maintain energy balance and physiological functions. Additionally, the liver is crucial in synthesis, including the production of proteins, glycogen, and bile, ensuring a balance in plasma components. Acting as a detoxification organ, the liver transforms toxic substances into relatively harmless compounds and eliminates them through excretory pathways. The storage and release of various physiologically active substances, such as vitamins and minerals, are also responsibilities of the liver. However, when the liver is subjected to external insults, such as viral infections, drug toxicity [1], or alcohol abuse, these essential functions may be compromised, leading to the occurrence of liver injury [2,3]. Once damage occurs, it can result in a range of health issues in the body. Liver damage is a common pathological condition in the early stages of various liver diseases, and persistent liver damage can lead to cirrhosis [4], liver fibrosis, hepatitis, and even liver cancer [5]. Due to its central position in the circulatory system, the liver is continually exposed to various chemical substances, rendering it susceptible to chemical liver damage. This constant exposure makes the liver more vulnerable to the potentially harmful effects of external environmental substances, thereby increasing the risk of chemical injury. Therefore, understanding the liver’s responses and protective mechanisms under such chemical exposure becomes particularly crucial. In this context, in-depth research into the liver’s adaptability and resistance reactions to different chemical compounds helps us gain a more comprehensive understanding of the mechanisms underlying chemical liver damage. Such insights provide a foundation for the development of corresponding protective strategies [6]. In recent years, with the rapid development of the industry and pharmaceuticals, the widespread use of chemical substances and excessive pollution emissions have become an undeniable issue. This has led to an increasing incidence of chemical-induced liver damage [7], making liver injury an escalating global health concern [8]. Various chemicals used in industrial production and pharmaceutical processes, including heavy metals, organic solvents, and pesticides, not only exist in the manufacturing of products but also enter the environment through waste discharge. These toxic substances accumulate over time, subjecting individuals to prolonged exposure and potential health threats. Therefore, the development of dietary supplements that can assist in protecting against chemical-induced liver damage prevent its occurrence and reduce the incidence of liver diseases becomes increasingly meaningful. Devoting efforts to the research and development of such supplements is not only poised to provide effective support for liver health but may also play a positive role in mitigating the adverse effects caused by chemical-induced liver injury. This endeavor holds promise for ensuring public health, enhancing the quality of life, and driving innovative developments in preventive strategies.

Polygonatum describes the dried rhizomes of *Polygonatum sibiricum* Delar. ex Redoute, a perennial herbaceous plant belonging to the genus Polygonatum Mill. in the Liliaceae family [9,10]. It is not only a nutritionally rich ingredient but also a traditional Chinese medicinal herb [11]. Polygonatum is highly praised for its outstanding nutritional value, primarily renowned for its various health benefits such as nourishing Qi and Yin, promoting spleen health, moisturizing the lungs, and tonifying the liver and kidneys. Regarded as a comprehensive nutritional supplement, Polygonatum is rich in beneficial natural elements and bioactive compounds, contributing to the maintenance of the body’s equilibrium and providing comprehensive nutritional support [12]. This popularity has positioned Polygonatum as one of the widely embraced herbal remedies, extensively utilized in traditional Chinese medicine practices for health and well-being. Through in-depth exploration in modern pharmacological research, Polygonatum has been found to possess a diverse and intricate array of pharmacological effects, showcasing extensive and multi-level impacts within the biological system, including liver protection [13], anti-cancer properties [14], anti-oxidant activity [15], prevention of osteoporosis [16], immune modulation [17], blood sugar control [18], cardiovascular protection [19], and enhancement of learning and memory abilities [20]. These studies not only unveil the versatility of Polygonatum but also delve into the potential mechanisms underlying its varied effects in different physiological and pathological processes, laying the foundation for further exploration of its applications in the medical field and health maintenance [21]. Polygonatum is a highly diverse plant, featuring a composition that encompasses a broad range of components, including but not limited to polysaccharides, saponins, flavonoids, resinous substances, amino acids, quinone compounds, vitamins, alkaloids, and various trace elements [22,23,24,25,26,27,28,29]. This comprehensive composition not only contributes to the esteemed status of Polygonatum in traditional herbal applications but also highlights its extensive potential in modern medicine and nutrition. The amalgamation of these elements underscores the recognition of Polygonatum for its multifaceted benefits, positioning it as a valuable resource with broad applications in both traditional and contemporary healthcare practices. Polygonatum polysaccharides are widely acknowledged as one of the crucial bioactive components found in this plant. However, despite some preliminary explorations, further comprehensive and systematic research is required to delve deeper into the pharmacological basis of Polygonatum polysaccharides. This is essential to thoroughly unravel their mechanisms of action within the biological system and to gain a more profound understanding of their potential medical applications. This study initially isolated water-soluble polysaccharides (PSPs) from heterophylla. Subsequently, through further purification steps, three homogeneous polysaccharide components were identified and designated as PSP-N-b-1, PSP-N-b-2, and PSP-N-c-1. This series of isolation, purification, and identification processes lay the groundwork for exploring the biological activities of these polysaccharides and their physiological and pharmacological roles in depth. The protective effects of these three polysaccharides on in vitro liver damage induced by CCL_4_ were investigated, providing robust experimental evidence for the development of natural polysaccharide dietary supplements and drugs for preventing and treating liver injuries. This not only contributes to a deeper understanding of the mechanisms by which polysaccharides protect liver health but also offers valuable references for future clinical applications and drug development. These research findings are expected to provide new directions for improving liver disease prevention and treatment strategies, thereby promoting scientific research and medical practices in related fields.

## 2. Results

### 2.1. Isolation and Purification of Polysaccharides

**Figure 1:**
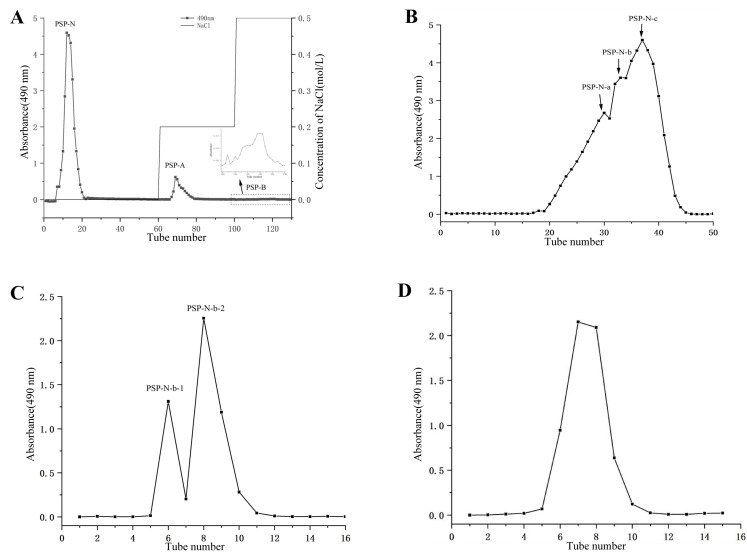
Elution curve of PSPs from DEAE-cellulose column (**A**), elution curve of PSP-N on Sepharose CL-6B gel (**B**), elution curve of PSP-N-b on Sephadex G100 gel (**C**), elution curve of PSP-N-c on Sephadex G100 gel (**D**).

Crude polysaccharides (PSPs) were obtained from Polygonatum by water extraction and alcohol precipitation methods. DEAE-cellulose ion exchange chromatography with a gradient elution (Figure 1A) was employed to obtain neutral sugar fraction PSP-N (67%), acidic sugar fraction PSP-A (3.4%), and PSP-B (0.4%). Further purification of PSP-N was performed by using Sepharose CL-6B (Figure 1B) to obtain three distinct elution peaks, which were named PSP-N-a (27.05%), PSP-N-b (11.25%), and PSP-N-c (18.5%) after lyophilization. The PSP-N-b and PSP-N-c fractions were purified again by using Sephadex G 100 (Figure 1C,D), Ultimately, PSP-N-b-1, PSP-N-b-2, and PSP-N-c-1 were obtained. The yields of PSP-N-b-1, PSP-N-b-2, and PSP-N-c-1 relative to crude astragaloside IV were 3.3%, 1.6%, and 6.6%, respectively.

### 2.2. Monosaccharide Composition and Molecular Weight

**Figure 2:**
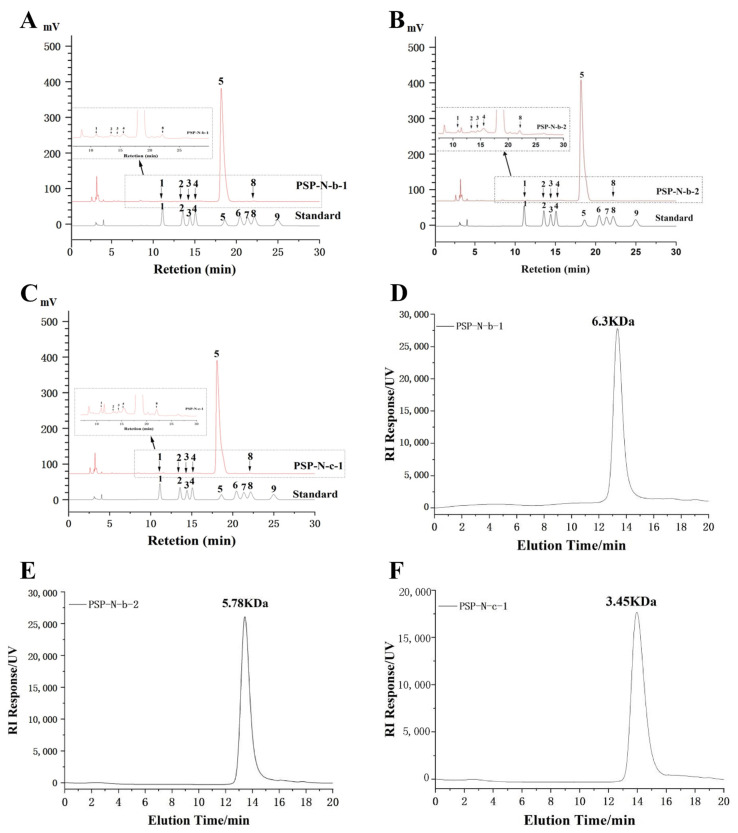
Monosaccharide composition and molecular weight determination results of each component of PSP-N. Monosaccharide composition: PSP-N-b-1 (**A**), PSP-N-b-2 (**B**), and PSP-N-c-1 (**C**); Molecular weights: PSP-N-b-1 (**D**), PSP-N-b-2 (**E**), and PSP-N-c-1 (**F**).

The three homogenized components were composed of different proportions of Man, GlcA, Rha, GalA, Glc, and Ara (Table 1, Figure 2A–C)).

Molecular weight determination of PSP-N-b-1, PSP-N-b-2, and PSP-N-c-1 was performed by using HPGPC.

PSP-N-b-1, PSP-N-b-2, and PSP-N-c-1, respectively, exhibit uniform molecular weights of 6.3 kDa, 5.78 kDa, and 3.45 kDa (Figure 2D–F).

### 2.3. Effects of PSPs and Their Fractions on CcL_4_-Induced Morphological Changes in HepG2 Cells

**Figure 3:**
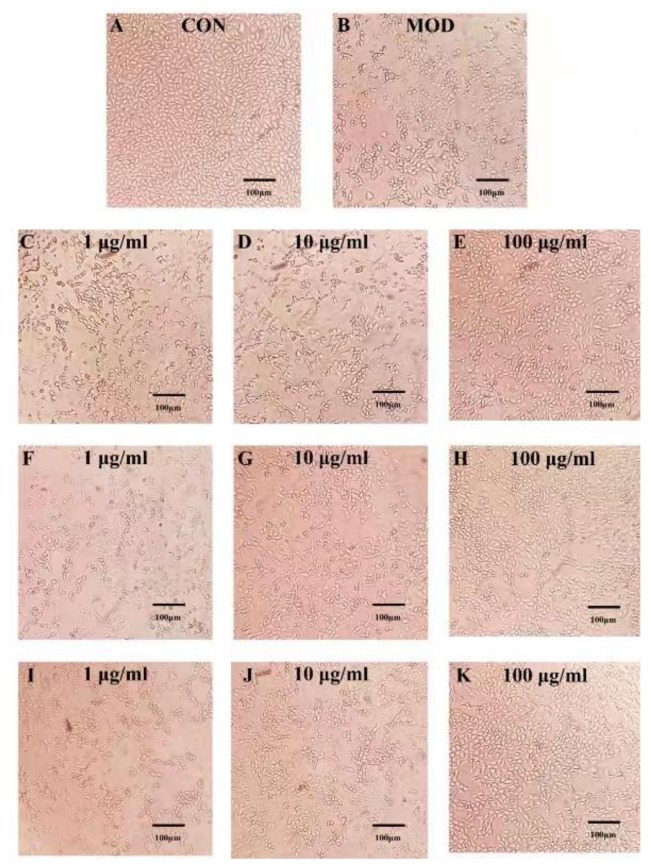
Effect of Polygonatum polysaccharides on the morphology of HepG2 cells induced by CCL_4_. Normal control group (**A**), model group (**B**), low-, medium-, and high-dose groups of PSP-N-b-1 (**C**–**E**), low-, medium-, and high-dose groups of PSP-N-b-2 (**F**–**H**), low-, medium-, and high-dose groups of PSP-N-c-1 (**I**–**K**).

The effect of Polygonatum polysaccharides on CcL_4_-induced HepG2 cells was shown in Figure 3. As observed under an inverted microscope, the HepG2 cells in the normal group had a good developmental status, with intact and mostly spindle-shaped or polygonal three-dimensional cell structures. However, the cells in the model group showed weakened three-dimensional feeling, reduced cell size, and morphological changes. As the drug concentration increased, the degree of cell shrinkage decreased, the structure gradually became clear, and the survival rate increased in each treatment group. The cell state was better than that of the model group, especially in the high-dose groups, which had significantly restored cell structures. Therefore, the cell status in each treatment group was better than that in the model group, and the effect was positively correlated with the drug concentration.

### 2.4. Hepatoprotective Effects of PSPs and Their Fractions against CcL_4_-Induced HepG2 Cell Toxicity

**Figure 4:**
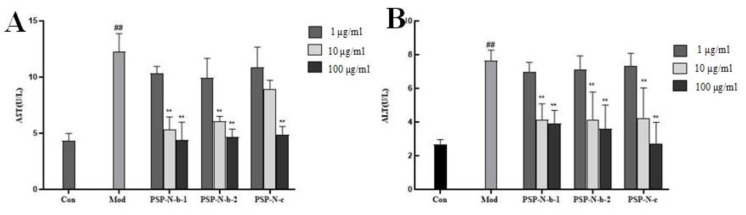
Effect of Polygonatum polysaccharides on ALT and AST of HepG2 induced by CCL_4_: AST (**A**), ALT (**B**). Compared with Con group, ## *p* < 0.01; compared with Mod group, ** *p* < 0.01.

The results of the ALT and AST activity measurements in the cell culture supernatant are shown in Figure 4. The ALT and AST activities in the Mod group were significantly higher than those in the Con group (*p* < 0.01), indicating the successful establishment of the model. Compared with the Mod group, different concentrations of crude polysaccharides from Polygonatum and its components were able to reduce the AST and ALT activities induced by CCL_4_ to varying degrees, and the differences were more significant at 10 μg/mL and 100 μg/mL (*p* < 0.01).

### 2.5. Effects of Polygonatum Polysaccharides on Oxidative Stress and Inflammatory Factors Induced by CCL_4_ in HepG2 Cells

**Figure 5:**
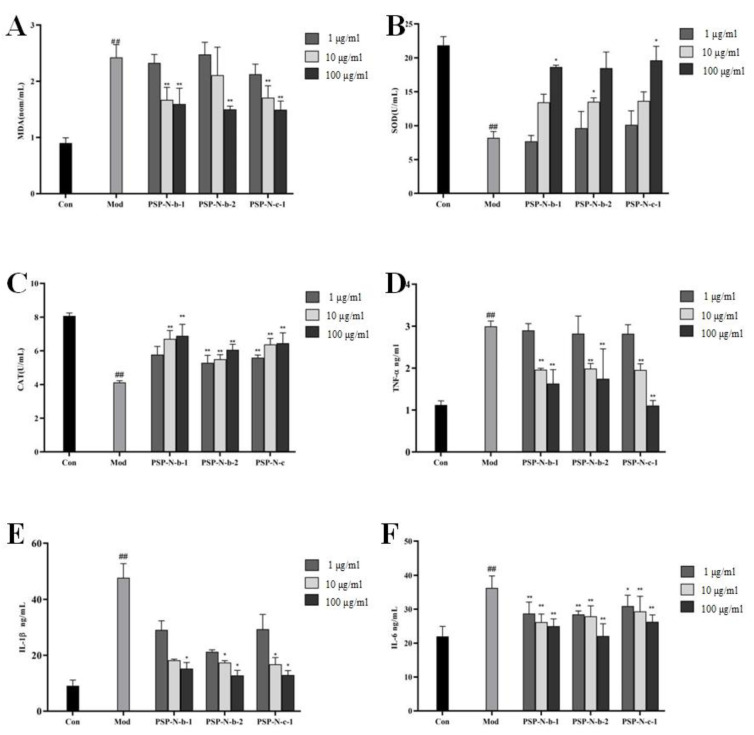
Effects of PSPs on oxidative indices and inflammatory cytokines in CCl_4_-induced HepG2 cells. MDA (**A**), SOD (**B**), CAT (**C**), TNF-α (**D**), IL-1β (**E**), and IL-6 (**F**). Compared with Con group, ## *p* < 0.01; compared with Mod group, * *p* < 0.05, ** *p* < 0.01.

The experimental results are shown in Figure 5. Compared with the Con group, the Mod group showed a significant increase in MDA content and a decrease in SOD and CAT content (*p* < 0.01). Compared with the Mod group, the MDA content decreased, while the SOD and CAT content increased in a dose-dependent manner. This indicates that Polygonatum polysaccharides exert liver-protective activity by clearing cellular free radicals and enhancing anti-oxidant capacity, and the differences were statistically significant (*p* < 0.05, *p* < 0.01).

As shown in Figure 5, compared with the Con group, the levels of TNF-α, IL-1β, and IL-6 in the Mod group increased significantly (*p* < 0.01). Compared with the Mod group, the levels of TNF-α, IL-1β, and IL-6 were significantly reduced in a dose-dependent manner, indicating that Polygonatum polysaccharides could effectively reduce the production of inflammatory factors and exert liver-protective activity. The differences mentioned above were statistically significant (*p* < 0.05, *p* < 0.01).

## 3. Discussion

Polysaccharides are natural macromolecules and polymers composed of monosaccharides linked by glycosidic bonds. Due to their predominant composition of carbon and oxygen, they are also commonly referred to as carbohydrates. Polysaccharides exhibit diverse structures and functions [30], and are typically formed by the linkage of two or more monosaccharide molecules through glycosidic bonds. These biological molecules are widely present in nature, being found in plants, animals, microorganisms, and other sources. Given their diversity, polysaccharides hold significant physiological and applied value in fields such as biology, medicine, and the food industry. The anti-oxidant properties of plant polysaccharides have been extensively documented in various studies [31]. These investigations indicate that plant polysaccharides exhibit significant anti-oxidant potential, capable of combating oxidative stress both internally and externally. Plant polysaccharides function as anti-oxidants by neutralizing free radicals, reducing oxidative damage, and modulating the balance of oxidation–reduction processes. As natural anti-oxidants, plant polysaccharides have garnered widespread attention and are considered to have substantial application potential in the biomedical and healthcare fields. In this study, the hepatoprotective effect of the homogeneous components of Polygonatum polysaccharides on CCL_4_-induced in vitro liver injury was evaluated [32,33].

The mechanism of CCL_4_-induced liver injury is mainly the generation of lipid peroxidation and inflammatory response [34]. CCL_4_ induces experimental liver injury by activating the cytochrome P-450-mediated generation of free radicals and reactive substances. Its hepatotoxicity primarily stems from the metabolic process that occurs after it enters the body, where CCL_4_ is metabolized into highly reactive trichloromethyl free radicals, directly impacting liver cells, and causing alterations in cell membrane structure and function. This process is initiated by activation of the cytochrome P-450 enzyme system within liver cells, ultimately leading to the occurrence of inflammation and, in severe cases, triggering cell death. Additionally, the entry of CCL_4_ into the body elicits an inflammatory response, promoting the production of inflammatory cytokines and chemokines, exacerbating the intensity of inflammation, and resulting in the proliferation of inflammatory cells in the damaged area. The entire process not only affects the structure and function of liver cells but also initiates an immune system response, culminating in a complex pathological progression that ultimately leads to experimental liver injury.

In this study, the oxidation indicators MDA, SOD, CAT, and inflammatory factors TNF-α, IL-1β, and IL-6 were measured in the supernatants of cell cultures. The results showed that in the Mod group, the MDA content increased while the SOD and CAT contents decreased, indicating the existence of lipid peroxidation in the cells. In the various treatment groups, the MDA content in the culture supernatant was lower than that in the Mod group, while the SOD and CAT contents were higher than those in the Mod group, indicating that the homogeneous components of Polygonatum polysaccharides can remove free radicals in cells and enhance the body’s anti-oxidant capacity. In addition, the levels of various inflammatory factors (TNF-α, IL-1β, IL-6) in the Mod group also significantly increased, indicating that the cells had undergone obvious inflammatory reactions, while the levels of TNF-α, IL-1β, and IL-6 in the various treatment groups significantly decreased, indicating that the homogeneous components of Polygonatum polysaccharides can effectively reduce the production of inflammatory factors. The results of the CCL_4_-induced experiments indicate that the homogeneous components of Polygonatum polysaccharides exhibit varying degrees of protective effects on cell viability and biochemical indicators in the supernatant(10 μg/mL–100 μg/mL). This suggests their potential protective efficacy in alleviating liver damage. This effect has a certain dose dependence, with the higher the dose (100 μg/mL) showing the better the protective effect. The various treatment groups exhibited differential effects on multiple indicators within distinct concentration ranges, suggesting that different components may possess varying degrees of hepatoprotective effects. The hepatoprotective activity of the three polysaccharide fractions (PSP-N-b-1, PSP-N-b-2, and PSP-N-c-1) were significantly higher than those of the unfractionated extract (PSP). Among them, PSP-N-c-1 had the strongest activity (see Figure S1). These differences in activity may be related to the difference in their molecular weight. In addition, further in vivo experiments are needed to substantiate the hepatoprotective effects of Polygonatum polysaccharides and their potential inhibitory effects on the pathways associated with liver damage. These in vivo experiments will contribute to a more in-depth confirmation of the hepatoprotective role of Polygonatum polysaccharides, elucidating their potential actions in suppressing pathways related to liver injury. Such in vivo experiments are crucial for pinpointing the exact mechanisms of action of Polygonatum polysaccharides and assessing their efficacy and safety in treating liver diseases. The systematic design and analysis of these experiments will provide robust support for subsequent clinical studies, laying a scientific foundation for the development of Polygonatum polysaccharides as a therapeutic agent for liver protection.

## 4. Materials and Methods

### 4.1. Materials and Equipment

The monosaccharide standard came from Sigma Alldrich Co. (St. Louis, MO, USA). Anhydrous ethanol was obtained from Tianjin Damao Chemical Reagent Factory (Tianjin, China). Phenol was purchased from Tianjin Guangfu Fine Chemical Research Institute (Tianjin, China). Concentrated sulfuric acid, CCL_4_, and NaCl were purchased from Beijing Chemical Plant (Beijing, China). Trypsin came from GENVIEW fetal bovine serum, which came from Hangzhou Sijiqing Company (Hangzhou, China). MTT was from Sigma Chemical Reagent Co., Ltd. in the United States. The 1640 culture medium came from Hyclone. DMSO and all analytical reagents (chromatographically pure) were obtained from Liaoning Quanrui Reagent Co., Ltd. (Liaoning, China). DEAE cellulose was purchased from Shanghai Yuanye Biotechnology (Shanghai, China). Agarose gel and CL-6B were purchased from Ruida Henghui Technology Development Co., Ltd. (Beijing, China). HepG2 cells were purchased from Beijing Dingguo Changsheng Biotechnology Co., Ltd. (Beijing, China). TNF-α, IL-1β, and IL-6 ELISA kits were purchased from Langdun Biotechnology Co., Ltd. (Foshan, China). AST, ALT, SOD, CAT, and MDA were purchased from Nanjing Jiancheng Bioengineering Institute (Nanjing, China).

HPLC came from Shimadzu International Trading Co., Ltd. (Kyoto, Japan). The Nuclear Magnetic Resonance was from Bruker Bio Spin, Fällanden, Switzerland. The Pure protein purification chromatography system was from GE Healthcare (Chicago, IL, USA). The UV visible spectrophotometer was from Shimadzu Corporation in Japan.

### 4.2. Isolation and Purification of Polysaccharides from Polygonatum

Polygonatum was purified by removing impurities, washing, drying, and cutting. The herb was extracted with distilled water at a ratio of 20:1 (*v*/*w*) at 80 °C for 2 h, for a total of 3 extractions. The supernatants were combined and filtered to remove small particles, and then concentrated using a rotary evaporator. The concentrated extract was mixed with 5 volumes of anhydrous ethanol and allowed to precipitate at 4 °C for 24 h. The precipitate was collected by centrifugation (3500 r, 5 min), washed with anhydrous ethanol, and then subjected to the Sevag method [35] (n-butanol: chloroform, 1:4 *v*/*v*) three times to remove proteins. The resulting polysaccharide solution was dialyzed against distilled water (MWCO 3500 Da, 48 h), concentrated, and freeze-dried to obtain crude polysaccharide powder, which was named PSP (8.36%).

Initially, the crude polysaccharide was dissolved in distilled water and applied to a pre-equilibrated DEAE-cellulose column (1.5 × 60 cm). The column was washed with distilled water, 0.2 M NaCl, and 0.5 M NaCl at a flow rate of 1 mL/min, and the eluted fractions were collected in 10 mL centrifuge tubes. Subsequently, fractions with similar results in the phenol–sulfuric acid method for total carbohydrates were combined to obtain PSP-N (distilled water), PSP-A (0.2 M NaCl), and PSP-B (0.5 M NaCl) fractions.

Further purification of the PSP-N fraction was performed using a Sepharose CL-6B column (2.6 × 100 cm) with a flow rate of 0.4 mL/min and elution with a 0.15 M NaCl solution. The eluted fractions were collected in 10 mL tubes, and fractions with similar properties were combined, freeze-dried, and named PSP-N-a, PSP-N-b, and PSP-N-c.

Lastly, additional purification of the PSP-N-b and PSP-N-c fractions was carried out using a Sephadex G100 chromatography column with distilled water as the eluent at a flow rate of 0.4 mL/min [36]. The eluted fractions were collected in 10 mL tubes, and fractions with similar properties were combined to obtain PSP-N-b-1, PSP-N-b-2, and PSP-N-c-1. This series of meticulous procedures ensured a high degree of purification and separation of the obtained products, laying a reliable foundation for subsequent experiments and analyses [37].

### 4.3. Molecular Weight Determination

Taking appropriate amounts of PSP-N-b-1, PSP-N-b-2, and PSP-N-c-1 polysaccharide samples, we dissolved them separately in a 0.2M NaCl solution to prepare a polysaccharide solution with a concentration of 5 mg/mL. After ensuring complete dissolution of the samples, we filtered them through a 0.22 μm filter membrane to eliminate suspensions and particles, ensuring the accuracy of subsequent chromatographic analysis. Next, we took 20 μL of the prepared polysaccharide solution and analyzed it by using high-performance gel permeation chromatography (HPGPC: Shimadzu LC-20AT system, RID-20A detector). We chose a TSK Gel G-4000PWXL column (7.8 × 300 mm), set the column temperature to 40 °C, used 0.2M NaCl as the mobile phase, and set the flow rate to 0.6 mL/min.

For polysaccharide molecular weight determination, a glucose polymer standard curve was prepared, including standard substances with molecular weights of 1 kDa, 5 kDa, 12 kDa, 25 kDa, and 50 kDa. The preparation of the standard curve should involve calibration and determination of the content and concentration of standard substances. In the actual measurement, precise determination of the molecular weight of the test polysaccharide was achieved by comparing the chromatographic peaks of the test sample with the corresponding peaks on the standard curve for each molecular weight.

### 4.4. Monosaccharide Composition Analysis

We precisely weighed an appropriate amount of polysaccharide, then dissolved it in a 2M hydrochloric acid–methanol solution to prepare a sample solution with a concentration of 2 mg/mL. The key was to ensure the complete dissolution of the polysaccharide to obtain a homogeneous experimental sample. Next, we inject it with nitrogen and placed the solution in a constant-temperature metal bath at 80 °C for a 16 h hydrolysis reaction. The choice of hydrolysis time and temperature were made to ensure the reaction reached maximum efficiency. After hydrolysis, we used a nitrogen blower to remove residual hydrochloric acid–methanol, ensuring the purity of the reaction product. Subsequently, transfer of the product to an environment containing 2M trifluoroacetic acid (TFA) and completion of a 1 h reaction at a high temperature (120 °C) to further transform the product and enhance reaction rate were performed. Following this, a thorough drying process was performed to ensure the sample was free of water, removing potential solvent residues and obtaining a dry and pure reaction product.

To further analyze and detect the reaction product, we employed 1-phenyl-3-methyl-5-pyrazolone (PMP) for pre-column derivatization to enhance the detection sensitivity and stability of the target product. Finally, we filtered the sample through a 0.22 μm membrane to remove any possible minute particles and impurities. Throughout the entire experimental process, we utilized a Shimadzu HPLC system, including an LC-20AT pump and SPD-20A UV-Vis detector, along with a COSMOSIL 5C18-PAQ column (specifications: 4.6 mm × 250 mm). The solvent conditions were 80.8% PBS (0.1M, pH 7.0) and 19.2% acetonitrile (*v*/*v*) in a 1:3 ratio for efficient detection of the target product.

### 4.5. Cell Culture

HepG2 cells were cultured in 1640 medium supplemented with 10% fetal bovine serum in a CO_2_ incubator set at 37 °C, saturated humidity, and 5% CO_2_ [38].

### 4.6. The Detection of Relevant Indicators

HepG2 cells were seeded into a 24-well plate with 500 μL of cell suspension per well. The cells were cultured for 24 h at 37 °C with 5% carbon dioxide. After discarding the supernatant, fresh culture medium was replenished, and each component was added according to the groups at concentrations of 1 μg/mL, 10 μg/mL, and 100 μg/mL. Blank wells contained complete medium only. After incubation, the supernatant from each well was collected, and the levels of ALT (alanine aminotransferase), AST (aspartate aminotransferase), MDA (malondialdehyde), SOD (superoxide dismutase), CAT (catalase), TNF-α (tumor necrosis factor-α), IL-6 (interleukin 6), and IL-1β (interleukin-1β) were determined following the instructions provided with the respective assay kits.

### 4.7. Data Processing

Statistical analysis of the experimental data was performed by using SPSS 19.0 software, and the results were expressed as mean ± standard deviation (x ± s). Inter-group comparisons were conducted using the *t*-test, and differences were considered significant when *p* < 0.05.

## 5. Conclusions

Three homogeneous fractions of Polygonatum polysaccharides were isolated from Polygonatum, each composed of different proportions of six monosaccharides: Man, GlcA, Rha, GalA, Glc, and Ara. The findings of this study underscore the diverse protective effects exhibited by all fractions against CCL_4_-induced damage in HepG2 cells. These effects are likely attributed to the anti-inflammatory and anti-oxidant mechanisms employed by the fractions, suggesting their potential therapeutic benefits in mitigating hepatocellular injury.

## Figures and Tables

**Table 1:** Monosaccharide composition of PSP fractions.

Title 1	Man	GlcA	Rha	GalA	Glc	Ara
PSP-N-b-1	0.4%	0.3%	0.3%	0.5%	98.1%	0.4%
PSP-N-b-2	0.3%	0.3%	0.3%	0.6%	98.3%	0.3%
PSP-N-c-1	0.5%	0.2%	0.3%	0.5%	98.1%	0.3%

## Data Availability

Data are contained within the article and Supplementary Materials.

## Supplementary Materials

The following supporting information can be downloaded at: https://www.mdpi.com/article/10.3390/molecules29051038/s1, Figure S1: Effects of Polygonatum polysaccharide and its homogeneous components on HepG2 cells induced by CCl_4_: AST (A), ALT (B), MDA (C), SOD (D), CAT (E), TNF-α (F), IL-1β (G), and IL-6 (H). Compared with Con group, ## *p* < 0.01; compared with Mod group, * *p* < 0.05, ** *p* < 0.01.
