# Supplementary material for: Isolation, Purification, Fractionation, and Hepatoprotective Activity of Polygonatum Polysaccharides
Source: Molecules. 2024 Feb 28;29(5):1038. doi: 10.3390/molecules29051038 (PMC10935244; doi:10.3390/molecules29051038)
Supplement: Supplementary file 1 [file molecules-29-01038-s001.zip › molecules-2855005-supplementary.pdf]

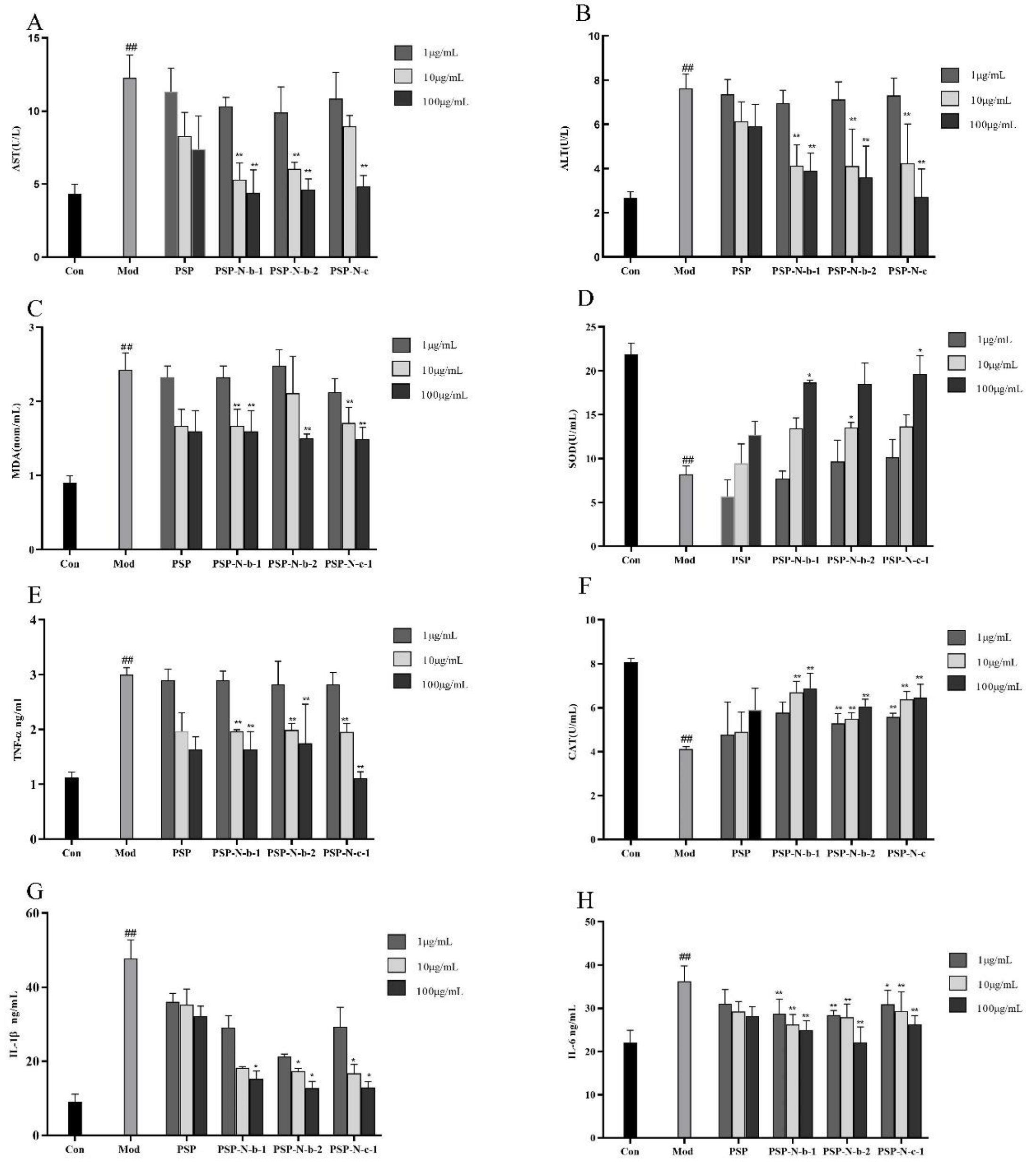

Figure S1 Effects of Polygonatum polysaccharide and its homogeneous components on HepG2 cells induced by CCl_4:AST (A), ALT (B), MDA (C) , SOD (D), CAT (E), TNF-α (F), IL-1β (G) , and IL-6 (H). Compared with Con group, $^{\#\#}P<0.01$; compared with Mod group, $^{*}p < 0.05$, $^{**}p < 0.01$.
